# Physiological and de novo transcriptome analysis of the fermentation mechanism of *Cerasus sachalinensis* roots in response to short-term waterlogging

**DOI:** 10.1186/s12864-017-4055-1

**Published:** 2017-08-22

**Authors:** Peng Zhang, Deguo Lyu, Luting Jia, Jiali He, Sijun Qin

**Affiliations:** College of Horticulture/Key Lab of Fruit Quality Development and Regulation of Liaoning Province, Shenyang Agricultural University, Shenyang, Liaoning 110866 People’s Republic of China

**Keywords:** *Cerasus sachalinensis*, Fermentation, Transcriptome, Waterlogging

## Abstract

**Background:**

*Cerasus sachalinensis* is widely used in cool regions as a sweet cherry rootstock and is known for its sensitivity to soil waterlogging and waterlogging stress. However, the limited availability of *Cerasus* genomic resources has considerably restricted the exploration of its waterlogging response mechanism. To understand its reaction to short-term waterlogging, we analyzed the physiology and transcriptomes of *C. sachalinensis* roots in response to different waterlogging durations.

**Results:**

In this study, 12,487 differentially expressed genes (DEGs) were identified from *Cerasus sachalinensis* roots under different waterlogging durations. Carbon metabolism and energy maintenance formed the first coping mechanism stage of *C. sachalinensis* in response to low oxygen conditions. Root energy processes, including root respiration and activities of the fermentation enzymes alcohol dehydrogenase, pyruvate decarboxylase, and lactate dehydrogenase, showed unique changes after 0 h, 3 h, 6 h, and 24 h of waterlogging exposure. Ribonucleic acid sequencing was used to analyze transcriptome changes in *C. sachalinensis* roots treated with 3 h, 6 h, and 24 h of waterlogging stress. After de novo assembly, 597,474 unigenes were recognized, of which 355,350 (59.47%) were annotated. To identify the most important pathways represented by DEGs, Gene Ontology and Kyoto Encyclopedia of Genes and Genomes databases were used to compare these genes. The first stage of root reaction to waterlogging stress was activation of carbohydrate metabolism to produce more glucose and maintain energy levels. At 3 h, the glycolytic and fermentation pathways were activated to maintain adenosine triphosphate production. At 24 h, pathways involved in the translation of proteins were activated to further assist the plant in tolerating waterlogging stress. These findings will facilitate a further understanding of the potential mechanisms of plant responses to waterlogging at physiological and transcriptome levels.

**Conclusions:**

Carbon metabolism and energy maintenance formed the first coping mechanism *C. sachalinensis* in response to low oxygen conditions, and they may be responsible for its short-term waterlogging response. Our study not only provides the assessment of genomic resources of *Cerasus* but also paves the way for probing the metabolic and molecular mechanisms underlying the short-term waterlogging response in *C. sachalinensis*.

**Electronic supplementary material:**

The online version of this article (doi:10.1186/s12864-017-4055-1) contains supplementary material, which is available to authorized users.

## Background

Soils with high clay content and poor drainage can be waterlogged or flooded by inappropriate irrigation practices or heavy rains. Under these conditions, excess water saturates the rhizosphere, and the remaining oxygen is quickly consumed by plant roots and soil microorganisms, resulting in hypoxic conditions [1]. In general, sweet cherry trees are grafted onto rootstocks that in turn determine the tolerance of the cherry tree to abiotic stress [2]. However, species of *Prunus* used as rootstocks are classified as sensitive to root hypoxia, although there are reported differences among genotypes regarding their ability to tolerate this stress [3, 4].

*Cerasus sachalinensis* (F. Schmidt) Kom. is native to northeastern China and northern Korea. This species is widely used as a sweet cherry rootstock in cool regions such as Dalian and Qinhuangdao because of its high propagation rate, cold resistance, and adaptability [5]. However, it is vulnerable to waterlogging after heavy rains, especially during the rainy season. Proper drainage systems are often lacking, and therefore waterlogging is very common for 3–24 h following rainfall. One study demonstrated that *C. sachalinensis* rootstocks are particularly sensitive to waterlogging [6]; the mechanism and responsive gene expression patterns, however, are not yet understood.

The diffusion rate of oxygen in water is much lower than in air, and therefore waterlogging inhibits plant growth and development, making the plants vulnerable to hypoxia or anoxia [7]. When roots are subjected to waterlogging, the oxygen-dependent energy-generating pathways (e.g., aerobic respiration) are suspended, which leads to a rapid reduction in cellular adenosine triphosphate (ATP). When the oxygen supply is limited, various adaptive responses are activated to address this energy depletion [8]. Some plants cope by relying on glycolysis and fermentation to supply essential ATP [9]. In other cases, plants that can survive under low oxygen conditions shift their energy metabolism from aerobic to anaerobic [10]. Plants that rely on fermentation pathways to regenerate nicotinamide adenine dinucleotide (NAD^+^) maintain the glycolysis pathway during these periods [11].

Cellular respiration both generates ATP, which is needed for cell maintenance and growth, and releases energy [12]. Waterlogging limits the oxygen supply, inhibits respiration, and greatly reduces the energy status of the roots. Respiration status, which reflects root physiological metabolic capacity, is often affected by biotic and abiotic factors, including waterlogging.

Changes in plant respiration pathways have been extensively studied. Approximately 20 anaerobically induced polypeptides (ANPs) have been identified [13]. ANPs are essential components for low oxygen tolerance in various plant species. Other studies have shown that ANPs were involved in the glycolytic and fermentation pathways that are necessary to maintain energy production under waterlogging conditions [14]. Subsequently, microarray studies have been conducted to assess responses to low oxygen [15, 16].

Ribonucleic acid sequencing (RNA-seq) provides a powerful tool for profiling the complete gene space of any organism owing to its high throughput, accuracy, and reproducibility. In plants, which often have large and complex genomes [17], RNA-seq has accelerated the discovery of novel genes, tissue-specific expression patterns, and functional analysis [18]. The RNA-seq approach has a higher sensitivity for gene expressions than microarrays. RNA-seq has been successfully used for waterlogging responses in rice [19], cucumber [20], maize [21], and rape [22]. However, only a few woody plants have been studied. To better understand the molecular mechanisms of the response of *C. sachalinensis* to waterlogging, the gene transcription changes from plants subjected to different durations of waterlogging were examined using the Illumina HiSeq™ 4000 sequencing platform (Illumina Inc., San Diego, CA, USA). The early stages of the response to waterlogging stress were focused on because they determine the switch from normal to low-oxygen metabolism and play an essential role in plant survival. Our results will facilitate understanding the response of waterlogging-intolerant woody plants to short-term waterlogging stress.

## Methods

### Plant growth and water treatments

*Cerasus sachalinensis* plants were obtained from Lianshanguang, Benxi, Liaoning Province, China (41°24′N, 124°17′E). Plants were grown in plastic pots (16 × 16 cm) under a transparent rain shelter at the experimental field (Shenyang, China, 41°N, 123°48′24″E) in April 2014. Over the course of the experiment, the average air temperature varied between 15 °C and 25 °C (mean = 20 °C ± 5 °C). Two weeks later, after seedlings had produced 10–12 leaves, 36 plants that were similar in height and free from disease were selected for the treatments. Plants were divided evenly into a control group (CK) and a treatment where pots were kept in tap water 3 cm deep (waterlogged, WL). WL roots were sampled at 3 h, 6 h, and 24 h after waterlogging was introduced, and CK roots were sampled at 0 h. The primary roots with some lateral roots were collected from each individual plant, frozen separately in liquid nitrogen, and stored at −80 °C. Roots were pooled prior to RNA extraction to prepare four samples: CK at 0 h, WL at 3 h, WL at 6 h, and WL at 24 h. All plants in the treatment (each treatment have nine plants) group were sampled at each time point.

### Root respiration

Roots were gently washed with deionized water and dried carefully with paper towels. Root samples of 0.05 g were used to measure respiration status as described by Zhou [5]. Root respiratory rate was measured as the oxygen consumption rate using an Oxytherm oxygen electrode (Hansatech, King’s Lynn, Norfolk, England) as reported by Bouma [23]. Root respiratory pathways were measured as described by Yu and Pan [24]. The contribution of each respiratory pathway was calculated with the following equation:

$$ \left[\left(\mathrm{total}\  \mathrm{respiration}\  \mathrm{rate}-\mathrm{residual}\  \mathrm{respiration}\  \mathrm{rate}\right)/\mathrm{total}\  \mathrm{respiration}\  \mathrm{rate}\right]\times 100\%. $$

### ATP content

The intracellular ATP content was measured using ATP determination kits (Nanjing Jiancheng Bioengineering Institute, Nanjing, Jiangsu province, China). The results were expressed as μmol ATP/g fresh weight.

### Analysis of total soluble sugars and starch

The concentrations of total soluble sugars and starch were analyzed by the anthrone method as described by Yemm and Willis [25]. Finely ground fresh tissue (approximately 150 mg) was homogenized in 3 mL of 80% ethanol and incubated in an ultrasonic bath for 30 min at 80 °C. After centrifugation (6000×*g*, 25 °C, 10 min), the supernatant was collected. The pellet was extracted again as described above, and the supernatant was obtained and combined with the previous aliquot. After adding 2 mL of anthrone reagent to the supernatant, the mixture was heated in boiling water for 10 min. After the mixture cooled to room temperature, the absorbance was measured at 620 nm. A standard curve was established using a series of diluted glucose solutions.

The pellet obtained after the extraction of the soluble sugars was further extracted with perchloric acid for starch determination. Subsequently, the starch (expressed as glucose equivalent) in the supernatant was determined with the same spectroscopic methods outlined above.

### Glycolytic and fermentative enzyme assays

Root samples (0.5 g per replicate) were immediately frozen in liquid nitrogen and stored at −80 °C for enzyme activity measurements as described previously [26]. Hexokinase (HK, EC 2.7.1.1), pyruvate kinase (PK, EC 2.7.1.40), pyruvate decarboxylase (PDC, EC 4.1.1.1), alcohol dehydrogenase (ADH, EC 1.1.1.1), and lactate dehydrogenase (LDH, EC 1.1.1.17) activities were measured at 340 nm using an ultraviolet spectrophotometer (Purkinje TU-1900, Beijing, China), as described previously [27]. The LDH assay was conducted in 1.5 mL of reaction mix that contained 50 mM potassium phosphate buffer (pH 7.0), 0.2 mM NADH, 3 μM potassium cyanide, 4 mM 4-methylpyrazole, 0.4 ml of sample, and 10 mM sodium pyruvate to initiate the reaction [28]. Protein concentration was measured according to the methods of Bradford [29].

### RNA extraction

RNA was extracted using the method of Chang [30], and then isolated and purified using a plant RNA extraction kit (R6827, Omega Bio-Tek, Norcross, GA, USA).

### RNA quantification and qualification

RNA was checked for contamination and degradation by running samples on a 1% agarose gel for 20 min at 150 mV. RNA purity was checked using a NanoPhotometer^®^ spectrophotometer (NanoDrop 2000, Thermo Fisher Scientific, Waltham, MA, USA). RNA concentration was measured using a Qubit^®^ RNA Assay Kit with the Qubit^®^ 2.0 Fluorometer (Life Technologies, Carlsbad, CA, USA). RNA integrity was assessed using the RNA Nano 6000 Assay Kit for the Agilent Bioanalyzer 2100 system (Agilent Technologies, Santa Clara, CA, USA).

### Library preparation for transcriptome sequencing

Complementary deoxyribonucleic acid (cDNA) library construction, sequencing, and assembly were performed following the methods of Su [31], Ma [32] and Wang [33]. Sequencing libraries were generated using a NEBNext® Ultra™ RNA Library Prep Kit for Illumina® (New England Biolabs, Ipswich, MA, USA), and index codes were added to attribute sequences to each sample. Messenger RNA (mRNA) was purified using poly-T oligo-attached magnetic beads. Fragmentation was carried out using divalent cations under elevated temperature in NEBNext First Strand Synthesis Reaction Buffer (5×). A random hexamer primer and M-MuLV Reverse Transcriptase (RNase H^−^) and DNA Polymerase I and RNase H were used to synthesize the first strand cDNA and second strand cDNA. After adenylation of 3′ ends of DNA fragments, a NEBNext Adaptor with a hairpin loop structure was ligated to prepare for hybridization. The library fragments were purified with the AMPure XP system (Beckman Coulter, Beverly, MA, USA). Then 3 μl USER Enzyme (New England Biolabs) was used with size-selected, adaptor-ligated cDNA at 37 °C for 15 min followed by 5 min at 95 °C. before the polymerase chain reaction (PCR), which was performed with Phusion High-Fidelity DNA polymerase, Universal PCR primers and Index (X) Primer. Finally, PCR products were purified (AMPure XP system) and library quality was assessed on the Agilent Bioanalyzer 2100 system. Illumina sequencing was performed at Novogene Bioinformatics Technology Co., Ltd., Beijing, China (www.novogene.com). The raw reads were deposited in the NCBI Sequence Read Archive (SRA, http://www.ncbi.nlm.nih.gov/Traces/sra).

### Gene annotation

The unigenes of the transcriptomes were annotated through comparison with public databases, including the National Center for Biotechnology Information (NCBI) nonredundant (NR) protein sequence database, the NCBI nucleotide (NT) sequence database, the eukaryotic ortholog group (KOG) database, the Kyoto Encyclopedia of Genes and Genomes (KEGG) ortholog (KO) database, the Swiss-Prot protein database, the Gene Ontology (GO) database, and the protein family (Pfam) database, using NCBI’s basic local alignment search tool (BLAST) with a cutoff E-value of 10^−5^.

### Quantification of gene expression levels and differential expression analysis

Gene expression levels were estimated using the software package RSEM for each sample as described previously [7]. The DEGseq (2010) R package was used to identify differential expression genes, with a q-value <0.05 and |log2 (fold change)| > 1 as the threshold for significant differential expression [34]. The GO seq R packages based on Wallenius’ noncentral hypergeometric distribution was used for GO enrichment analysis [35]. KOBAS software was used to test the statistical enrichment of differentially expressed genes (DEGs) in KEGG pathways [36].

### qPCR analysis

The expression patterns of twelve genes that encode enzymes involved in the glycolysis/gluconeogenesis pathway, such as *c271205_g2* (phosphofructokinase), *c275115_g1*, *c275115_g3*, and *c249838_g1* (pyruvate decarboxylase), *c261927_g6* (glucose-6-phosphate isomerase), *c251063_g2* (LDH), *c253776_g1* and *c272347_g1* (ADH), *c268143_g3* (PK), *c249865_g1* (superoxide dismutase), and *c240800_g1* (L-ascorbate peroxidase), were analyzed using quantitative PCR (qPCR). New plant material was used for the RNA extraction for the qPCR assays. Three biological replicates were analyzed. Gene-specific primers were designed with Primer Premier 5.0 software. The reaction mixture included 5 μl SYBR Green Premix Ex Taq II (DRR820A, Takara, Dalian, China), 0.8 μl primes, 2.4 μl ddH_2_O, 1 μl cDNA and was run in an ABI StepOne™ Plus system. The 2^-ΔΔT^ method was used to analyze the relative expression levels of genes.

### Statistical analyses

All statistical analyses were performed with PASW software (version 18.0, SPSS, IBM, Armonk, NY, USA). The mean values of enzyme activities and root respiration rates between treatments were analyzed by one-way analysis of variance (ANOVA) and compared for statistically significant differences as determined by Duncan’s multiple range test (*p* < 0.05).

## Results

### Energy status of waterlogged roots

**Fig. 1:**
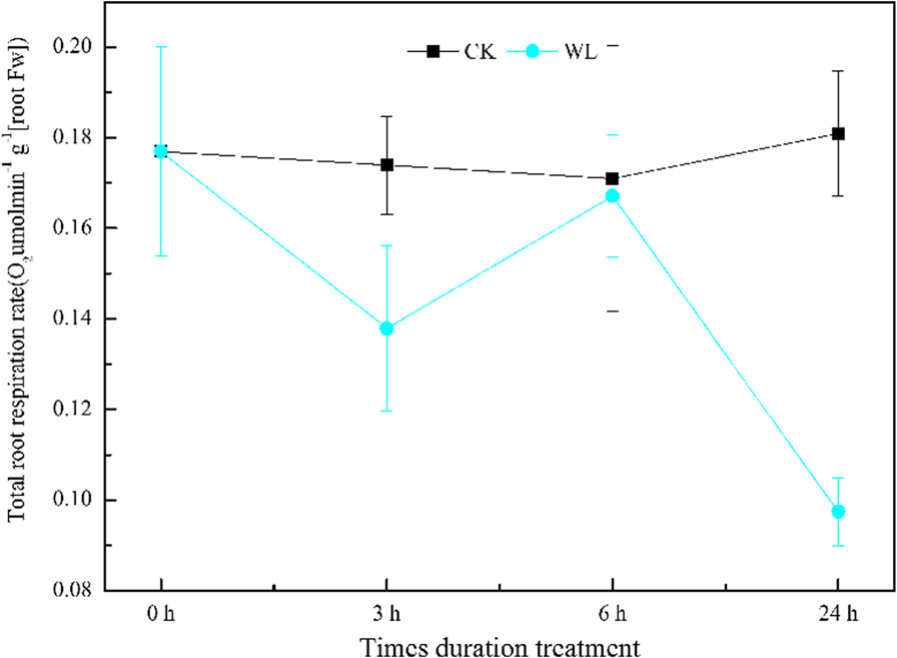
Root respiration rate in *Cerasus sachalinensis* roots at different waterlogging durations. Note: Data indicate means (*n* = 9) ± SD

**Fig. 2:**
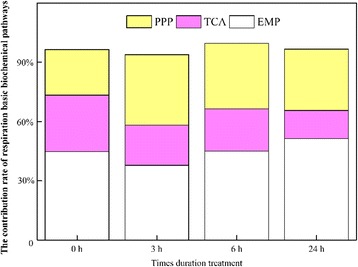
The contribution of basic respiration biochemistry pathways in *Cerasus sachalinensis* roots at different waterlogging durations

To characterize energetic responses to waterlogging in *C. sachalinensis*, we monitored the root respiration rate, contribution from root respiration pathways, and ATP content. In our study, the respiration rates decreased by 21% and 56% after 3 h and 24 h of waterlogging treatment, respectively, but no changes were observed at 6 h (Fig. 1). After 3 h, the basic respiration pathways changed from Embden-Meyerhof-Parnas (EMP)-tricarboxylic acid (TCA)-pentose phosphate pathway (PPP) to EMP-PPP-TCA. As the duration of waterlogging increased, the contribution rate of EMP decreased by 6.90% at 3 h and increased by 0.30% and 6.60% at 6 h and 24 h, respectively, compared to the control. The contribution rate of TCA was the lowest, ranging from 29.39%–14.21%; the contribution rate of PPP increased by 12.54%, 9.54%, and 7.89% at 3 h, 6 h, and 24 h, respectively (Fig. 2).

**Fig. 3:**
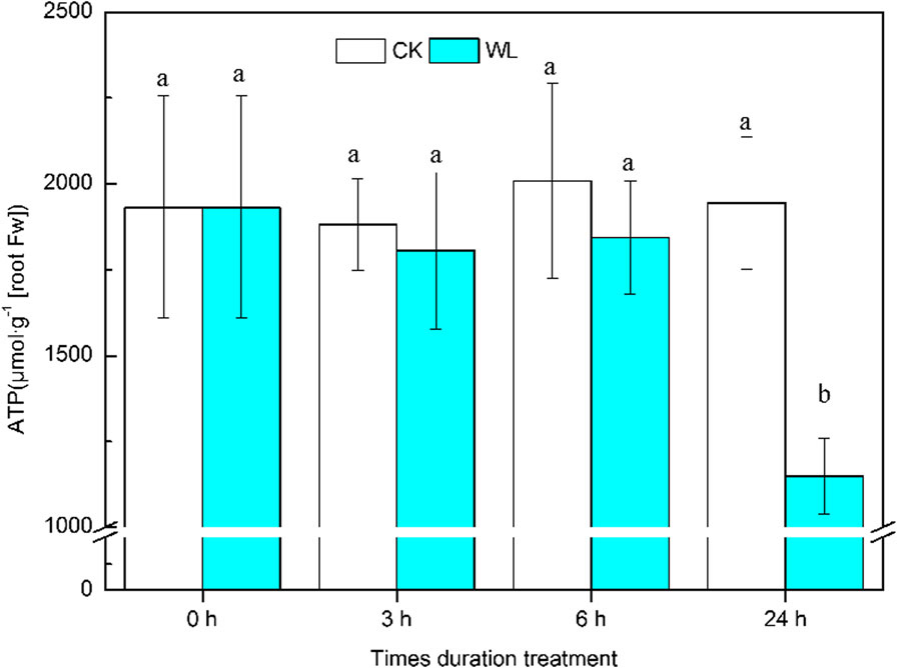
ATP content in *Cerasus sachalinensis* roots at different waterlogging durations. Note: Data indicate means (*n* = 9) ± SD. Different letters denote significant differences among means judged by a one-way ANOVA in relation to the control (Duncan’s multiple range test, *p* < 0.05)

ATP is used as an energy source in root cells, and therefore its content can reflect the energy status of roots. There were no significant differences in treated roots at 3 h and 6 h, but at 24 h, ATP content had significantly decreased (*p* < 0.05) (Fig. 3).

**Fig. 4:**
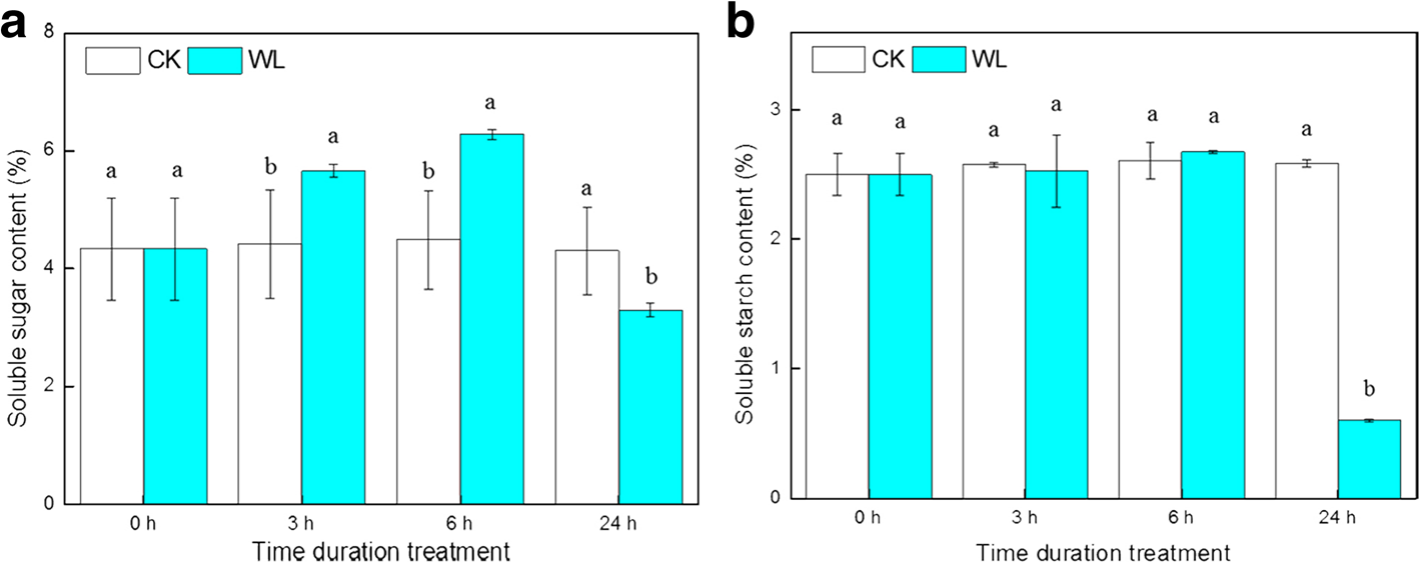
Carbohydrate concentration in *Cerasus sachalinensis* roots at different waterlogging durations. Note: Data indicate means (*n* = 9) ± SD. Different letters denote significant differences among means judged by a one-way ANOVA in relation to the control (Duncan’s multiple range test, *p* < 0.05). **a** soluble sugar content; **b** soluble starch content

Concentrations of total soluble sugars and starch were examined during waterlogging (Fig. 4). In general, the high mean concentrations of total soluble sugars showed no significant differences at 0 h, 3 h, and 6 h, but significantly decreased at 24 h. Starch concentrations increased at 3 h and 6 h, but decreased at 24 h.

### Glycolytic and fermentative enzymes

**Fig. 5:**
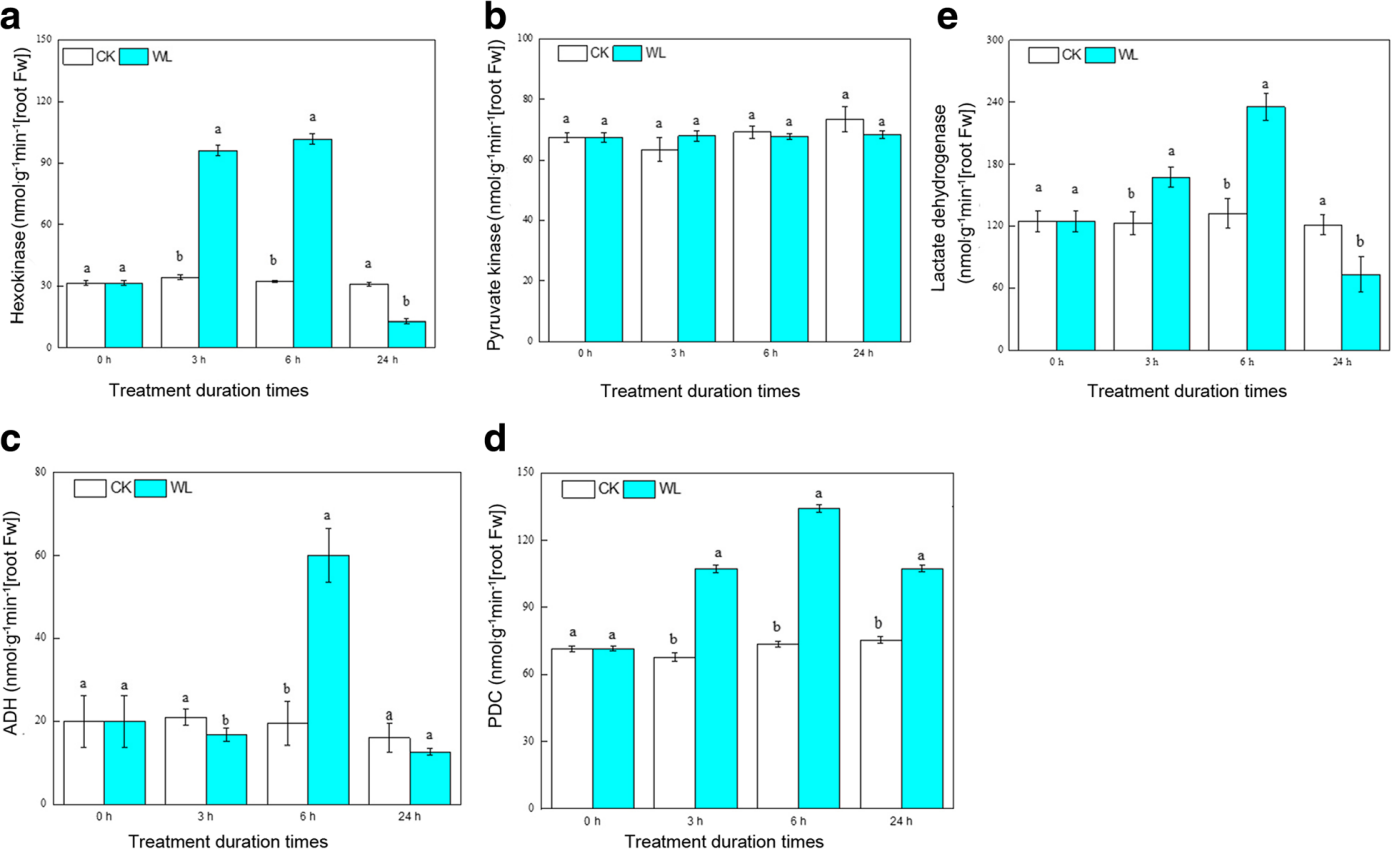
Changes in glycolytic and fermentative enzymes at different waterlogging times in *Cerasus sachalinensis* roots. Note: Data indicate means (*n* = 9) ± SD. Different letters denote significant differences among means judged by a one-way ANOVA in relation to the control (Duncan’s multiple range test, *p* < 0.05). **a**–**e** activity of hexokinase, pyruvate kinase, alcohol dehydrogenase, pyruvate decarboxylase, and lactate dehydrogenase in control and waterlogging treatments

To investigate the role of glycolysis and fermentation in mitigating energy deficits, we measured the activities of five enzymes: PK, HK, LDH, ADH, and PDC (Fig. 5). In *C. sachalinensis* roots, the activity of PK, one key enzyme of the EMP pathway, was not affected by waterlogging, but the activity of HK, another important enzyme, increased significantly at 3 h and 6 h, and then decreased at 24 h. The activity of LDH, the key enzyme for lactic acid fermentation, increased greatly at 3 h and 6 h, and then decreased at 24 h. At 3 h and 24 h, the activity of ADH, a key enzyme in ethanol fermentation, was not significantly different from the control, but at 6 h, it increased significantly (*p* < 0.05). Compared to the control, the activity of PDC, another central enzyme for ethanol fermentation, was significantly higher after 3 h, 6 h, and 24 h of waterlogging, and increased by 1.59-, 1.82-, and 1.42-fold, respectively.

### Sequence annotation and coding sequence prediction

Transcriptome sequences and the Illumina assembly data from *C. sachalinensis* roots were deposited in the NCBI Sequence Read Archive database under accession number SRP108195. In total, 617,203,902 paired-end raw reads were generated (Table 1). After adaptor sequences, ambiguous nucleotides, and low-quality sequences were removed, there were 588,477,556 clean reads remaining. Clean reads were assembled into 597,474 unigenes of 201–16,708 bp with an N50 length of 601 bp (Additional file 1: Figure S1). In total, 462,668 unigenes (77.43%) were between 200 and 500 bp; 79,121 unigenes (13.24%) were between 501 and 1000 bp; 37,855 unigenes (6.34%) were between 1001 and 2000 bp; and 17,830 unigenes (2.98%) were longer than 2000 bp (Additional file 2: Figure S2).

**Table 1:** Summary of sequences analysis in *Cerasus sachalinensis* roots

Sample	Raw Reads	Clean reads	Clean bases	Error(%)	Q20(%)	Q30(%)	GC(%)
CK_0h_1	46,798,272	44,621,416	6.69G	0.02	95.18	89.01	46.95
CK_0h_2	45,107,014	43,159,672	6.47G	0.02	95.31	89.26	46.96
CK_0h_3	45,874,828	44,106,108	6.62G	0.02	95.5	89.54	46.63
WL_3h_1	54,417,084	52,087,158	7.81G	0.02	95.14	88.93	45.63
WL_3h_2	45,655,674	43,708,280	6.56G	0.02	95.1	88.87	45.74
WL_3h_3	53,722,372	51,549,180	7.73G	0.02	95.34	89.27	46.09
WL_6h_1	53,385,772	50,952,524	7.64G	0.02	95.05	88.81	46.39
WL_6h_2	57,430,360	54,787,486	8.22G	0.02	94.91	88.53	45.75
WL_6h_3	53,239,000	51,043,882	7.66G	0.02	95.34	89.3	46.96
WL_24h_1	49,054,868	46,282,834	6.94G	0.02	95.52	89.59	46.34
WL_24h_2	57,973,622	54,699,464	8.2G	0.03	94.52	87.62	47
WL_24h_3	54,545,036	51,479,552	7.72G	0.02	95.32	89.23	46.3
Sumary	617,203,902	588,477,556	88.26G				

Q20: The percentage of bases with a Phred value >20

Q30: The percentage of bases with a Phred value >30

*GC* GC content

To understand the function of the assembled transcripts, the unigenes were annotated through comparison with entries in seven public databases (Table 2). Analyses showed that 228,046 unigenes (38.2%) had significant matches in the NR database, 149,145 (25.0%) in the NT database, and 226,826 (38.0%) in the Swiss-Prot database. In total, 355,350 unigenes (59.47%) were successfully annotated in at least one of the seven databases, with 35,167 unigenes (5.88%) in all databases.

**Table 2:** BLAST analysis of nonredundant unigenes against public databases

	Number of Unigenes	Percentage (%)
Annotated in NR	228,046	38.16
Annotated in NT	149,145	24.96
Annotated in KO	105,551	17.66
Annotated in SwissProt	226,826	37.96
Annotated in PFAM	234,997	39.33
Annotated in GO	241,843	40.47
Annotated in KOG	142,876	23.91
Annotated in all Databases	35,167	5.88
Annotated in at least one Database	355,350	59.47
Total Unigenes	597,474	100

**Fig. 6:**
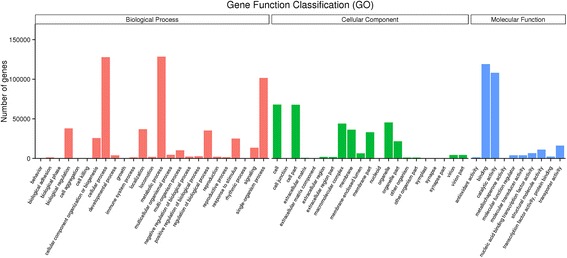
Gene ontology classifications of 241,843 orthologous unigenes

For GO analysis, 241,843 unigenes were divided into three ontologies (Fig. 6). In the biological process category, genes involved in metabolic (128,475), cellular (127,807), and single-organism (102,113) processes were well represented. The cellular component category was mainly comprised of proteins involved in the cell (67,949), cell parts (67,898), and organelles (45,395). Within the molecular function category, binding (119,125), catalytic activity (108,077), and transporter activity (16,235) were highly represented. In addition, assembled unigene functions were evaluated through a search against the KOG database for functional prediction and classification.

**Fig. 7:**
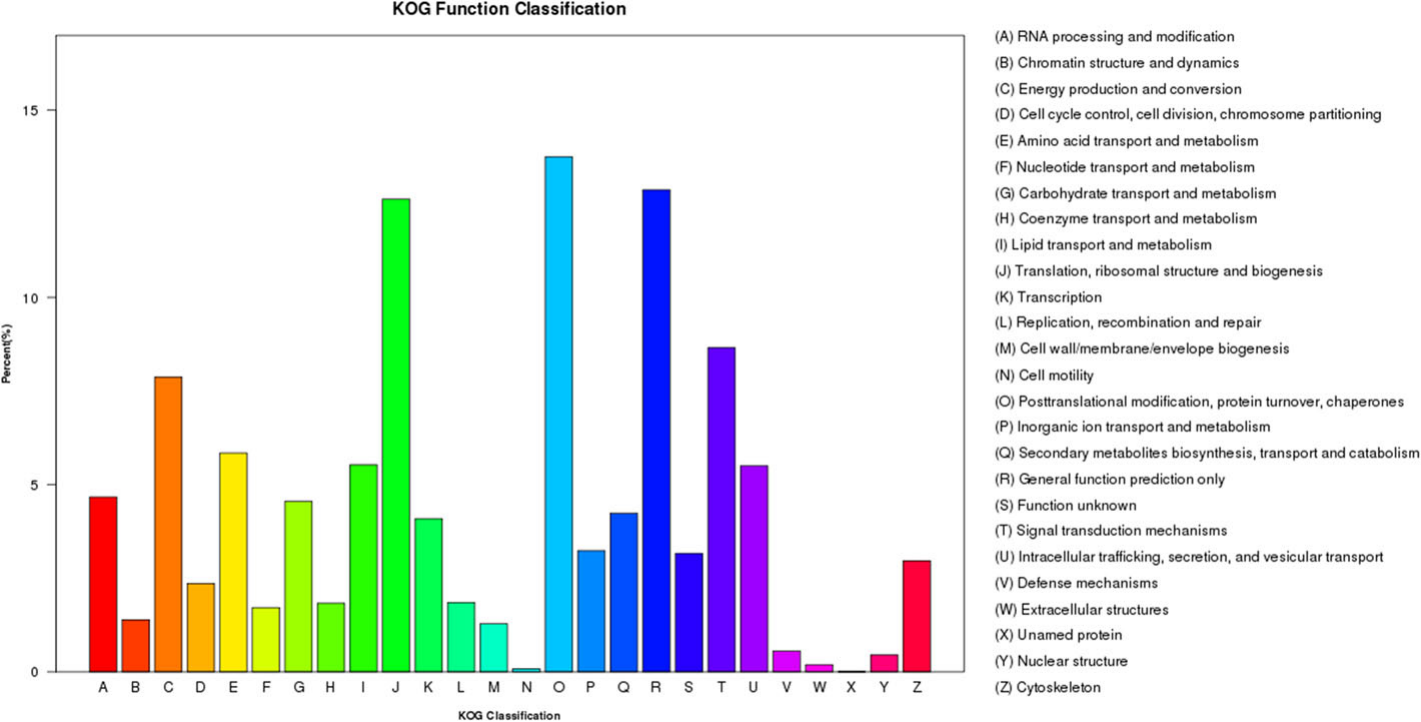
KOG annotation of unigenes

In all, 142,876 unigenes were assigned to a KOG classification and divided into 26 specific categories (Fig. 7). The largest group was post-translational modification, protein turnover, and chaperones (19,668); followed by general function prediction only (18,401); translation, ribosomal structure, and biogenesis (18,046); signal transduction mechanisms (12,367); and energy production and conversion (11,258). Only a few unigenes were assigned to the extracellular structures (271) and cell motility (114) categories.

**Fig. 8:**
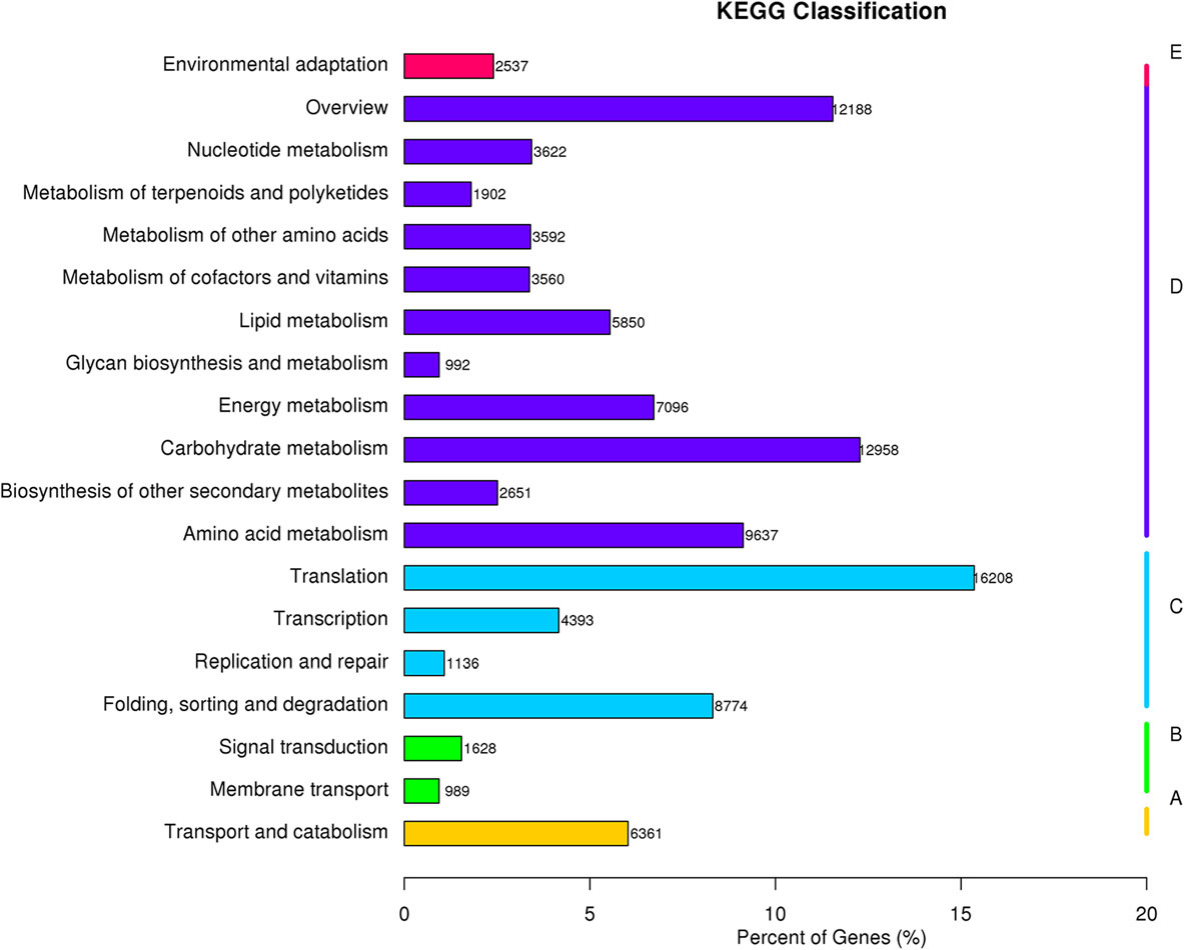
KEGG annotation of unigenes

The unigene metabolic pathway analysis was conducted using the KEGG annotation system. This process predicted 132 pathways with 135,260 unigenes (Fig. 8). The pathways involving the highest numbers of unique transcripts were translation (16,208), carbohydrate metabolism (12,958), amino acid metabolism (9637), folding, sorting, and degradation (8774), and energy metabolism (7096).

### Differential expression analysis of assembled *C. sachalinensis* transcripts from waterlogging stress

Using our de novo assembled transcriptome as a reference, we identified transcriptional responses to waterlogging. Reads from samples that had been exposed to waterlogging stress (3 h, 6 h, and 24 h) and from the control (0 h) were mapped to the obtained nonredundant unigenes from four libraries. The mapped reads were then used to estimate transcription levels according to fragment per kilobase of transcript per million reads (FPKM) values. On average, 74.54% of the clean reads were mapped (Table 1).

**Fig. 9:**
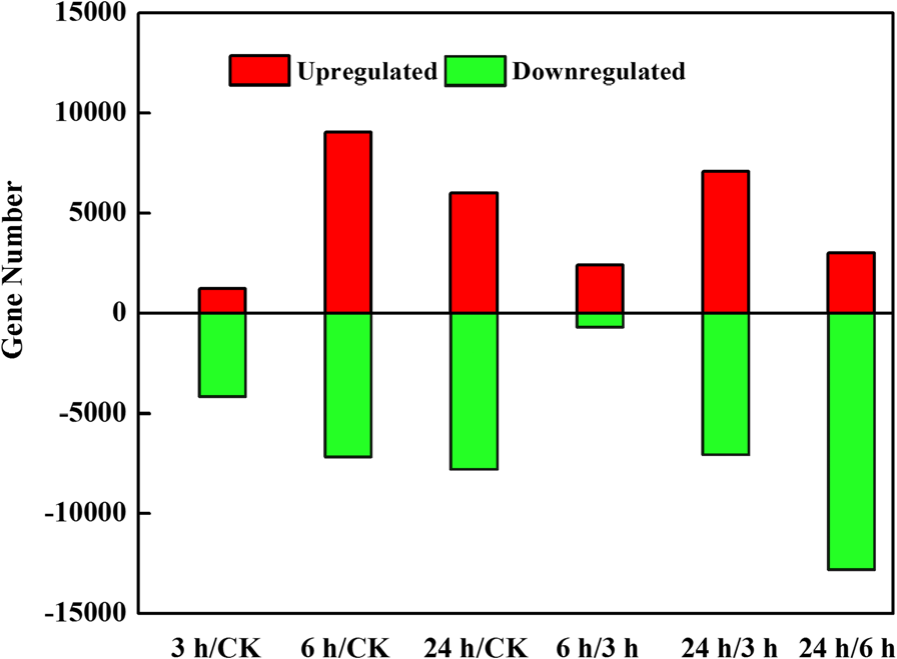
Transcriptomes of *Cerasus sachalinensis* roots under waterlog stress. **a** Number of unigenes expressed in each treated sample; (**b**) Number of differentially expressed genes showing up- (*red*) or down- (*green*) regulation between two-time points (DEGSeq, q < 0.05)

The genes with a q-value <0.05 and |log2 (foldchange)| > 1 were identified as significantly enriched or depleted. In total, 12,487 out of 597,474 unigenes (2.1%) were identified as DEGs among the treatments. The program DEGseq was used to identify the DEGs between the waterlogged and control samples (3 h/0 h, 6 h/0 h, and 24 h/0 h, respectively). DEGs with higher expression levels were considered upregulated (16,261), whereas those with lower expression levels were downregulated (19,101) (Fig. 9), that means some genes were up or down at different time points.

**Fig. 10:**
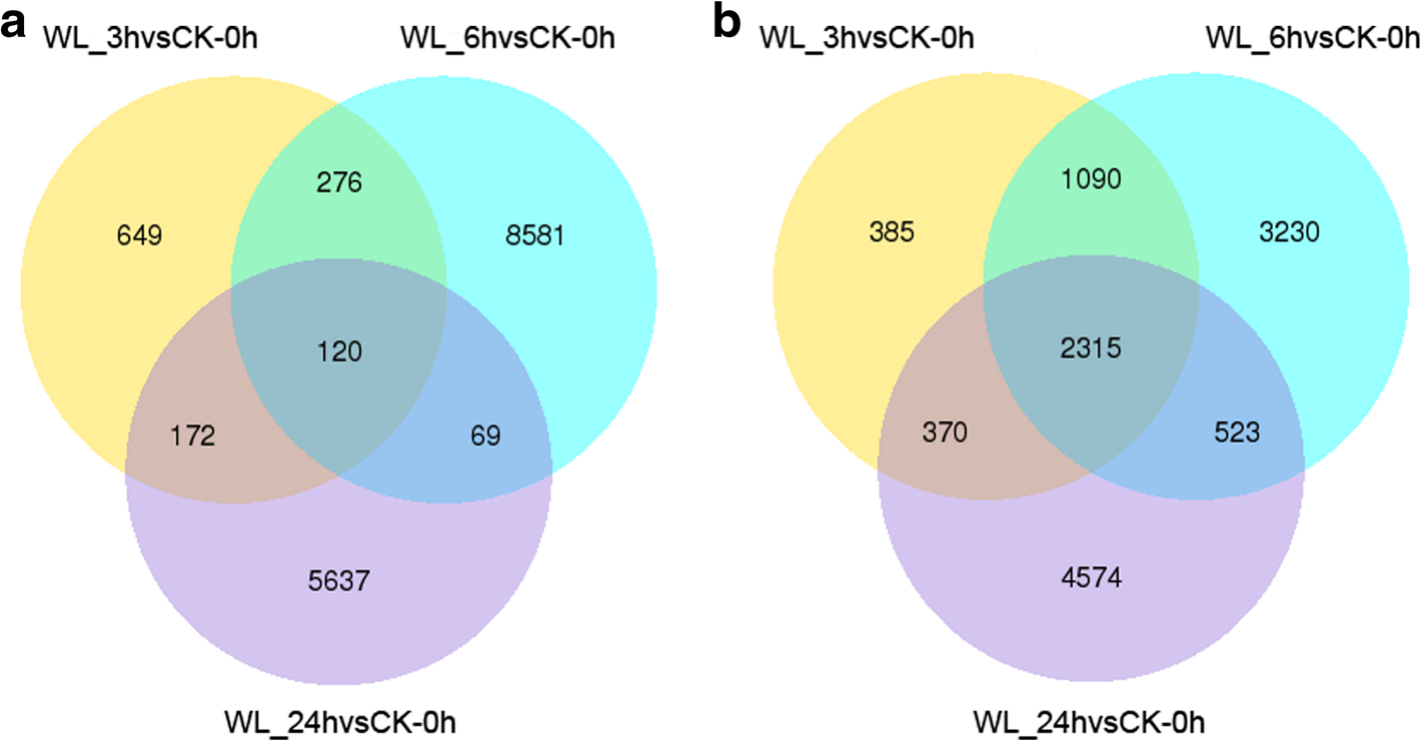
Venn diagrams of the differential expression transcripts under different treatment times. The number of DEGs exclusively up- or downregulated in *Cerasus sachalinensis* roots is shown. The number of DEGs with a common or opposite tendency of expression change between the two waterlogging times is shown in the overlapping regions. **a** Venn diagrams of the upregulated differential expression transcripts; **b** Venn diagrams of the downregulated differential expression transcripts

Compared with the control, 1217 genes were upregulated and 4160 were downregulated at 3 h. At 6 h, 9046 were upregulated and 7158 were downregulated, and at 24 h, 5998 were upregulated and 7783 were downregulated. Among these DEGs, 2447 were differentially expressed among all three treatments. In addition, 120 upregulated and 2327 downregulated genes were detected in all treatments (Fig. 10).

### Functional classification of DEGs

To further analyze the possible functions of DEGs, we conducted a GO enrichment analysis for DEGs with the entire transcriptome as the background and compared each pair of samples.

GO enrichment analysis of DEGs at 3 h compared to the control indicated that certain genes related to processes such as gluconeogenesis were overexpressed. mRNAs that were highly enriched (q ≤ 0.05, after false discovery rate correction) at 3 h encoded proteins involved in organic substance biosynthetic, biosynthetic, primary metabolic, carbohydrate metabolic, and organic substance metabolic processes, suggesting that genes involved in these processes may play important roles in the initial response to waterlogging. GO enrichment analysis at 6 h indicated that the waterlogging treatment may have inhibited energy-consuming biosynthetic processes, with the top five overexpressed genes linked to the biological process category: regulation of cellular process, biological regulation, regulation of biological process, transcription, and DNA-templated and nucleic acid-templated transcription. All of these were linked to the process that controls ATP consumption. At 24 h, ribosome biogenesis, ribonucleoprotein complex biogenesis, translation, peptide biosynthetic process, and peptide metabolic process appeared to play important roles in waterlogging responses.

Compared with the control, KEGG pathway enrichment analysis for DEGs indicated that five pathways (carbon fixation in photosynthetic organisms, glycolysis/gluconeogenesis, nitrogen metabolism, plant-pathogen interaction, and starch and sucrose metabolism) were significantly enriched, and only proteasome was significantly depleted at 3 h (q ≤ 0.05) (Additional file 3: Table S1). At 6 h, the flavonoid biosynthesis pathway was significantly enriched, and the proteasome and spliceosome pathways were significantly depleted (q ≤ 0.05). At 24 h, the top four enriched pathways were ribosome, monoterpenoid biosynthesis, oxidative phosphorylation, and glycolysis/gluconeogenesis. Only the diterpenoid biosynthesis pathway was significantly depleted.

### Response to waterlogging stress

We identified a relationship between gene expression and the activities of enzymes. We found that these pathways were significantly affected by waterlogging stress. The glycolysis/gluconeogenesis pathway was significantly enriched during the 24 h of waterlogging. There were 233 unigenes annotated as encoding enzymes involved in the glycolysis/gluconeogenesis pathway, and most of them were upregulated (Additional file 4: Table S2).

Most genes associated with sucrose metabolism were upregulated under waterlogging stress. Genes related to sucrose synthase were upregulated compared to the control; However, the expression of invertase genes were downregulated, which might explain the progressive increase in sucrose (Additional file 5: Figure S3)

The regulation of glycolysis indicates that ATP production may occur during different phases of waterlogging stress. Each glucose-1-P molecule is oxidized to L-lactate or ethanol, producing two ATP molecules. Most genes associated with fermentation were upregulated under waterlogging stress.

**Fig. 11:**
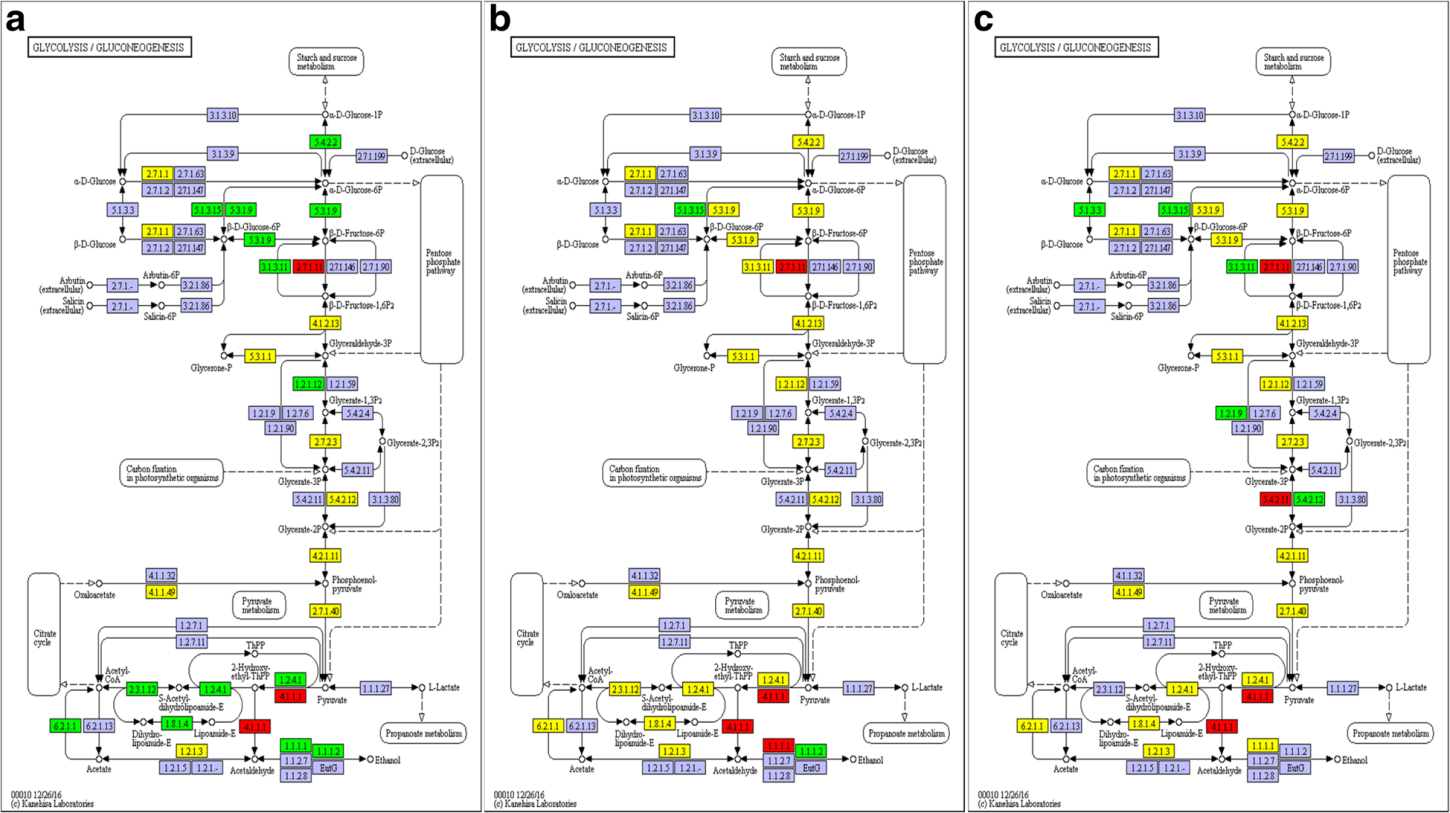
Unigenes predicted to be involved in the glycolysis pathway. Red indicates significantly increased expression compared with the control (CK); green indicates significantly decreased expression; yellow indicates proteins encoded by both up- and downregulated genes. **a** 3 h/CK; **b** 6 h/CK; **c** 24 h/CK; purple indicates no significantly changed

Two DEGs annotated as encoding PDC, *c275115_g3* and *c275115_g1*, had 3.21- and 2.77-fold increased expression, respectively, at 3 h compared to the control. *c253776_g1*, which encodes ADH, was downregulated 8.93-fold, which may have led to a lower ATP level in the root at 3 h (Fig. 11). At 6 h, three DEGs annotated as encoding PDC, *c275115_g3*, c*275115_g1*, and *c249838_g1*, had 3.8-, 3.13-, and 6.83-fold increased expression, respectively. *c251063_g2*, which encodes LDH, was increased 2.90-fold, and *c272347_g1*, which encodes ADH, increased 10.20-fold, indicating that the fermentation pathway was activated to maintain ATP production in waterlogged *C. sachalinensis* roots under hypoxic conditions. At 24 h, *c275115_g3* and *c275115_g1*, which encode PDC, were increased 4.15- and 4.51-fold.

### qPCR validation

To verify the credibility of the RNA-seq data, the transcriptional levels of 12 unigenes, most of them not only associated with glycolysis and fermentation but also with different expression levels, were examined by real-time quantitative PCR (Additional file 6: Table S3). The results supported the RNA-seq data (Additional file 7: Figure S4, Additional file 8: Figure S5).

## Discussion

Dynamic changes in mRNAs and metabolites in response to low oxygen conditions have been evaluated in a wide range of species, including *Arabidopsis thaliana* (L.) Heynh. [37], rice [19], mung bean [38], *Chlamydomonas reinhardtii* P. *A. dang*. [39], *Jatropha curcas* L. [40], and kiwifruits [41]. The metabolic changes found in our study were similar to those found in these species. Sasidharan [38] demonstrated that waterlogging caused elevated levels of mRNAs that encode the enzymes LDH, PDC, and ADH. Similarly, we found that mRNAs encoding enzymes involved in fermentation were also changed in *C. sachalinensis* roots under waterlogging conditions.

### Comparisons of metabolism and transcriptomes among different waterlogging durations

*Cerasus sachalinensis* is one of the most popular rootstocks in northeastern China and is known for its sensitivity to waterlogging [6]. At present, little genomic information is available for *Cerasus,* and the response of *Cerasus* to waterlogging stress has not been investigated through transcriptome analysis.

Metabolic responses to waterlogging varied during different periods of the experiment. At 3 h, compared to the control, the root respiration rate, especially the rate of the TCA cycle, decreased in response to waterlogging, whereas the glycolytic flux and relatived enzyme activities maintained a relatively high level. By 6 h, the roots enacted metabolic changes to cope with waterlogging, and normal metabolic activities under these adverse conditions were ensured by a high soluble carbohydrate concentration and glycolysis-related enzyme activities. With persistent waterlogging conditions at 24 h, soluble carbohydrates were nearly depleted and ATP generation was inhibited, resulting in an energy crisis in the waterlogged roots.

The transcriptome differences were compared among different waterlogging times and indicated 12,487 DEGs. Among them, 5377 were found at 3 h, 16,204 were found at 6 h, and 13,780 were found at 24 h. Thus, we concluded that 6 h as a peak of transcriptional changes in four times. These findings are similar to those of Liu, who studied *Arabidopsis* roots [42]. In contrast, the research on *Prunus avium* (L.) L. Mazzard F12/1, a rootstock that is sensitive to hypoxia, showed that after 6 h of hypoxic root conditions, only 764 DEGs changed their expression and 65% of DEGs were upregulated; the greatest difference in the number of DEGs was observed at 72 h of hypoxia [43].

To identify pathway changes among varying waterlogging durations, we compared the enriched KEGG pathways at different times (Additional file 9: Figure S6). Compared with that of the control, the response at 3 h showed that carbohydrate-related and energy-related mRNAs were abundant, presumably to maintain glycolytic flux in the root. After 6 h of waterlogging, biosynthetic process pathways, and in particular flavonoid biosynthesis, were significantly enriched (q < 0.05), likely because flavonoid accumulation plays a role in the removal of radical oxygen species (ROS) [44]. After 24 h, translation, metabolism of terpenoids and polyketides, energy metabolism, and carbohydrate metabolism were enriched. The responses of plant roots to waterlogging stress differed according to the duration of waterlogging. Initially, carbohydrate-related and energy-related DEGs were upregulated to provide energy generation at 3 h. Next, because ROS accumulated as the byproducts of metabolic processes, the flavonoid biosynthesis pathway was enriched to remove ROS at 6 h. Finally, as the waterlogging time lengthened and energy depletion became a greater concern, the pathways that benefited from the maintenance of cellular energy generation were enriched.

### Effects of waterlogging on carbohydrate metabolism

Carbohydrates are the primary energy resource under waterlogging conditions, and they are an important nutrient for hypoxic roots. Soluble sugars and starch are important carbohydrates; previous studies have shown that sufficient soluble sugar reserves were essential for plant survival during waterlogging. Research on mung bean [38] found that waterlogging-tolerant genotypes retained higher contents of sugar than intolerant genotype. In addition, research on two species of *Rorippa* found that 1 d of submergence significantly reduced the sugar concentration [45]. Short-term waterlogging in *Lotus corniculatus* L. var. *japonicus* Regel resulted in increased starch concentrations, whereas the total soluble sugar concentrations were not significantly changed [46]. Research on cucumber found that starch metabolism was activated after 8 h of waterlogging [20], which was similar to our study in which after 3 h of waterlogging, not only the carbohydrate concentration but also the genes involved in carbohydrate metabolism, including the glycolysis/gluconeogenesis pathway and starch metabolism, were affected in the roots. However, after 24 h of waterlogging, *C. sachalinensis* showed a rapid decrease in starch and soluble sugar concentrations, which distinguished the reaction in this species from that of other plants.

### Effect of waterlogging on fermentation pathways

Under low oxygen waterlogging conditions, oxygen-dependent root respiration is greatly limited. Glycolysis is one of the important pathways for energy production under hypoxic conditions, and maintaining enhanced glycolysis may be crucial for tree survival [47]. In our research, glycolysis was significantly enriched at 3 h and 24 h.

The glycolysis and fermentative processes were enhanced under waterlogging conditions. Fermentative processes included ethanol fermentation (catalyzed by PDC and ADH) and lactate fermentation (catalyzed by LDH). ADH accelerates ethanol fermentation and allows for glycolysis to supply the plants with ATP during waterlogging, which improves the ability of plants to acclimate to stress [48]. ADH and LDH are primarily involved in anaerobic metabolism, whereas PDC is involved in both anaerobic and aerobic metabolism.

Research on *Dendranthema* spp. [49], *Arabidopsis thaliana* [50], and cucumber [51] showed that waterlogging enhanced both the transcript abundance of *ADH*, *LDH*, and *PDC* and the activity of the three enzymes. Some studies have shown that under waterlogging conditions, lactic acid fermentation was activated first, followed by alcoholic fermentation [7, 47]. In gray poplar, LDH transcripts rapidly increased in abundance after 5 h of hypoxic treatment but dropped after 24 h of hypoxia because of low cytosolic pH [52]. Research has shown that ethanol fermentation was the dominant energy conversion process [53, 54], and therefore most studies focused on *ADH* and *PDC*. In *Arabidopsis thaliana*, whereas four genes encode PDC, only *PDC1* and *PDC2* were upregulated under anoxia [55]. Komatsu [56] reported at least six *ADH* genes, but only the *ADH2* gene was specifically expressed under waterlogging. However, other research found that *ADH1* was induced shortly after the onset of waterlogging [57, 58].

In our research, LDH activity increased significantly after 6 h of waterlogging and then decreased after 24 h (*p* < 0.05). PDC was the key enzyme that linked glycolysis and fermentation, and its levels increased rapidly after 3 h of waterlogging, whereas ADH and LDH activities were significantly increased in *C. sachalinensis* roots (*p* < 0.05). ADH was encoded by at least two genes that showed different expressions at different times. One *ADH* gene (c253776_g1) was significantly downregulated at 3 h, another *ADH* gene (c272347_g1) was significantly upregulated at 6 h (*p* < 0.05), and both PDC genes were also significantly upregulated. These results indicated that although the two *ADH* genes (c253776_g1 and c272347_g1) were strongly induced, they played different roles during waterlogging. Lactate and alcohol fermentation were activated in *C. sachalinensis* roots at 6 h, thereby marking 6 h as a peak of metabolic and transcriptional changes.

## Conclusion

In this study, transcriptomes of *C. sachalinensis* roots were sequenced using the Illumina platform. In total, 588,332,548 high-quality reads with 88.3 Gb sequence coverage were obtained, 597,474 unigenes (≥ 200 bp) were assembled, and 59.47% were annotated.

Transcripts involved in carbon metabolism changed under different waterlogging durations. In *C. sachalinensis*, these changes were mediated by a shortage of ATP. The glycolysis and fermentation pathways were stimulated to maintain ATP, and as a result, anaerobic respiration was high at 6 h. Thus, transcript patterns revealed that energy maintenance was the primary coping mechanism that *C. sachalinensis* adopted to survive under low oxygen conditions, which may be responsible for its remarkable waterlogging sensitivity.

We compared transcriptome differences among roots that were waterlogged for varying durations. In total, 12,487 DEGs were identified. Additionally, through comparisons to the control (3 h/0 h, 6 h/0 h, and 24 h/0 h), genes responsive to waterlogging were determined. There were 120 transcripts that showed upregulation and 2315 that were downregulated in all three pairwise comparisons between the waterlogged and control samples. In this transcriptome analysis, we found that energy maintenance was the primary coping mechanism under short-term waterlogging conditions. Six hours was an important time at which there was a peak in metabolic and transcriptional changes. These results will contribute to elucidating the metabolic and molecular mechanisms for short-term waterlogging in species sensitive to such adverse conditions.

## Additional files

Additional file 1: Figure S1. Length distribution of assembled unigenes. (TIFF 2478 kb)

Additional file 2: Figure S2. Length distribution of unigenes and transcript in *Cerasus sachalinensis* roots. (PPT 109 kb)

Additional file 3: Table S1. Significantly enriched metabolic pathways at different waterlogging durations. (XLSX 12 kb)

Additional file 4: Table S2. Differentially expressed genes of glycolysis/gluconeogenesis pathway at different waterlogging durations. (XLSX 97 kb)

Additional file 5: Table S3. Primers for RT-qPCR of all tested genes. (XLSX 12 kb)

Additional file 6: Figure S3. Changes in the expression of genes in sucrose and fermentation pathways of *C. sachalinensis* under waterlogging stress. (PPT 540 kb)

Additional file 7: Figure S4. Real-time PCR validation of the tested genes expression. (PPT 352 kb)

Additional file 8: Figure S5. Correlation of gene expression results respectively obtained by two methods (RT-qPCR analysis and RNA-Seq). (TIFF 7672 kb)

Additional file 9: Figure S6. Scatter plot of KEGG pathway enrichment statistics and the most enrichment pathway at different waterlogging durations. a-f: Top 20 statistics of up-regulated and down regulated pathway enrichment at different waterlogging durations. (PPTX 2794 kb)

## Abbreviations

ADH: Alcohol dehydrogenase
ANOVA: Analysis of variance
ANPs: Anaerobically induced polypeptides
ATP: Adenosine triphosphate
cDNA: Complementary deoxyribonucleic acid
CK: Control
DEG, DEGs: Differentially expressed gene(s)
FPKM: Fragments per kilobases per million mapped reads
GO: Gene ontology
HK: Hexokinase
KEGG: Kyoto Encyclopedia of Genes and Genomes
KO: Kyoto Encyclopedia of Genes and Genomes ortholog database
KOG: Eukaryotic ortholog group
LDH: Lactate dehydrogenase
mRNA: Messenger RNA
NAD: Nicotinamide adenine dinucleotide
NCBI: National Center for Biotechnology Information
NR: NCBI nonredundant protein sequences
NT: NCBI nonredundant nucleotide sequences
PCR: Polymerase chain reaction
PDC: Pyruvate decarboxylase
Pfam: Protein family
qPCR: Quantitative PCR
RNA: Ribonucleic acid
RNA-seq: High-throughput RNA-sequencing
WL: Waterlogged treatment
