# Supplementary figures and images for: Physiological and de novo transcriptome analysis of the fermentation mechanism of Cerasus sachalinensis roots in response to short-term waterlogging
Source: BMC Genomics. 2017 Aug 22;18:649. doi: 10.1186/s12864-017-4055-1 (PMC5568329; doi:10.1186/s12864-017-4055-1)

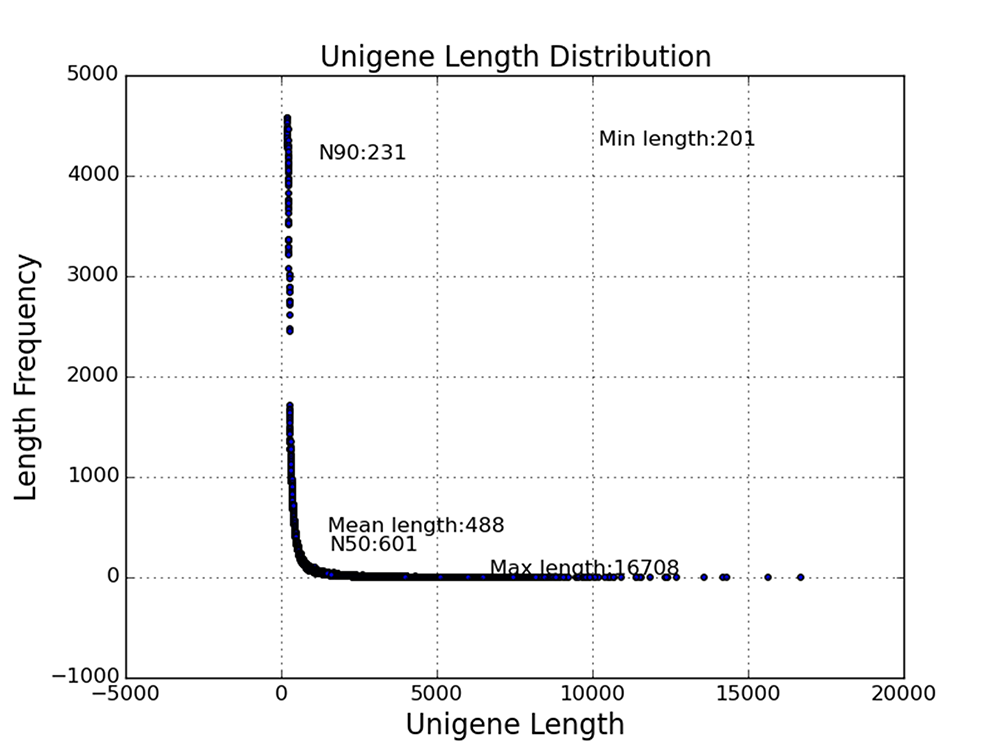

Supplement: Supplementary file 1 — Length distribution of assembled unigenes. (TIFF 2478 kb) [file 12864_2017_4055_MOESM1_ESM.tif]

## Slide 1
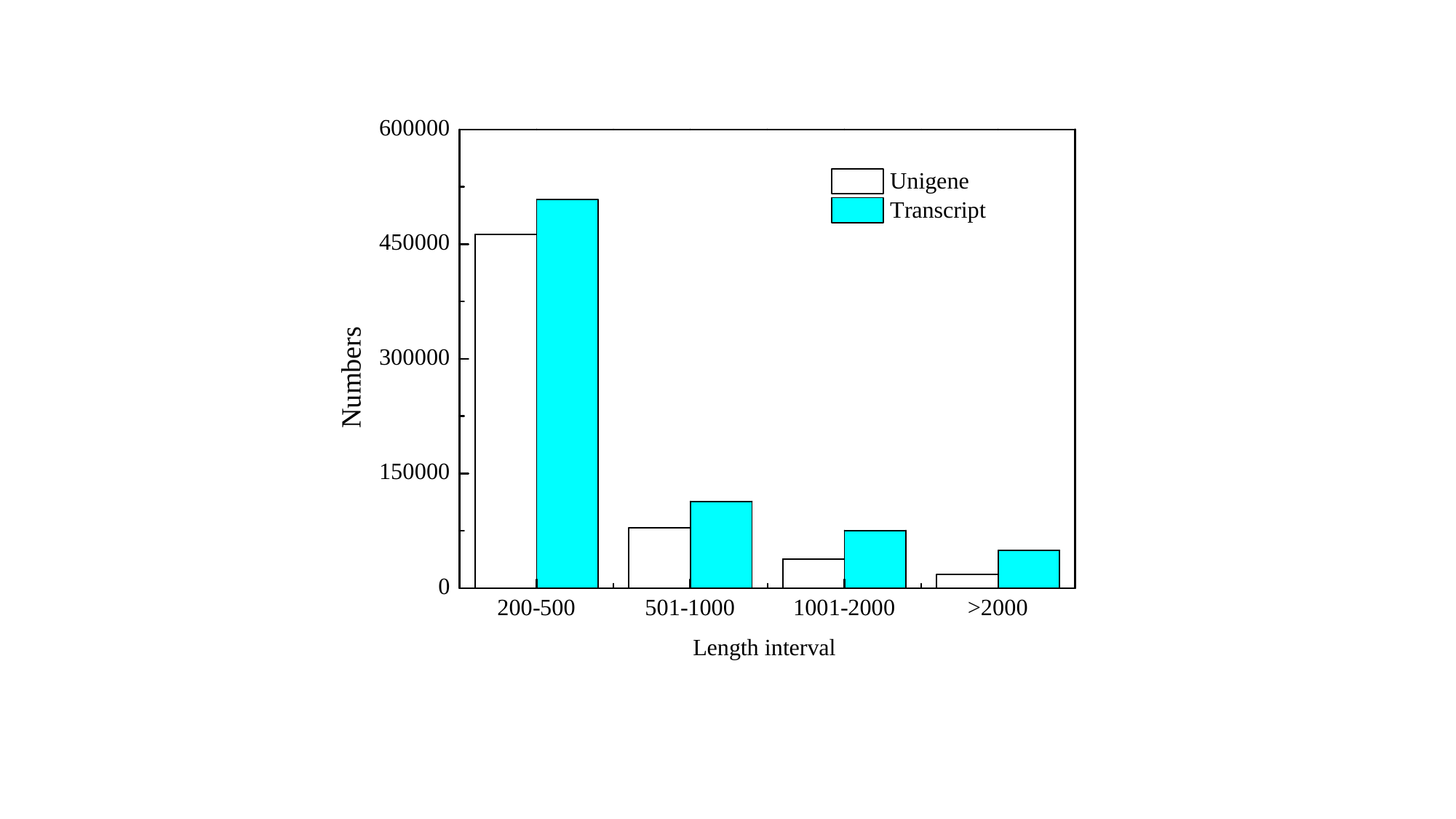

Supplement: Supplementary file 2 — Length distribution of unigenes and transcript in Cerasus sachalinensis roots. (PPT 109 kb) [file 12864_2017_4055_MOESM2_ESM.ppt]

## Slide 1
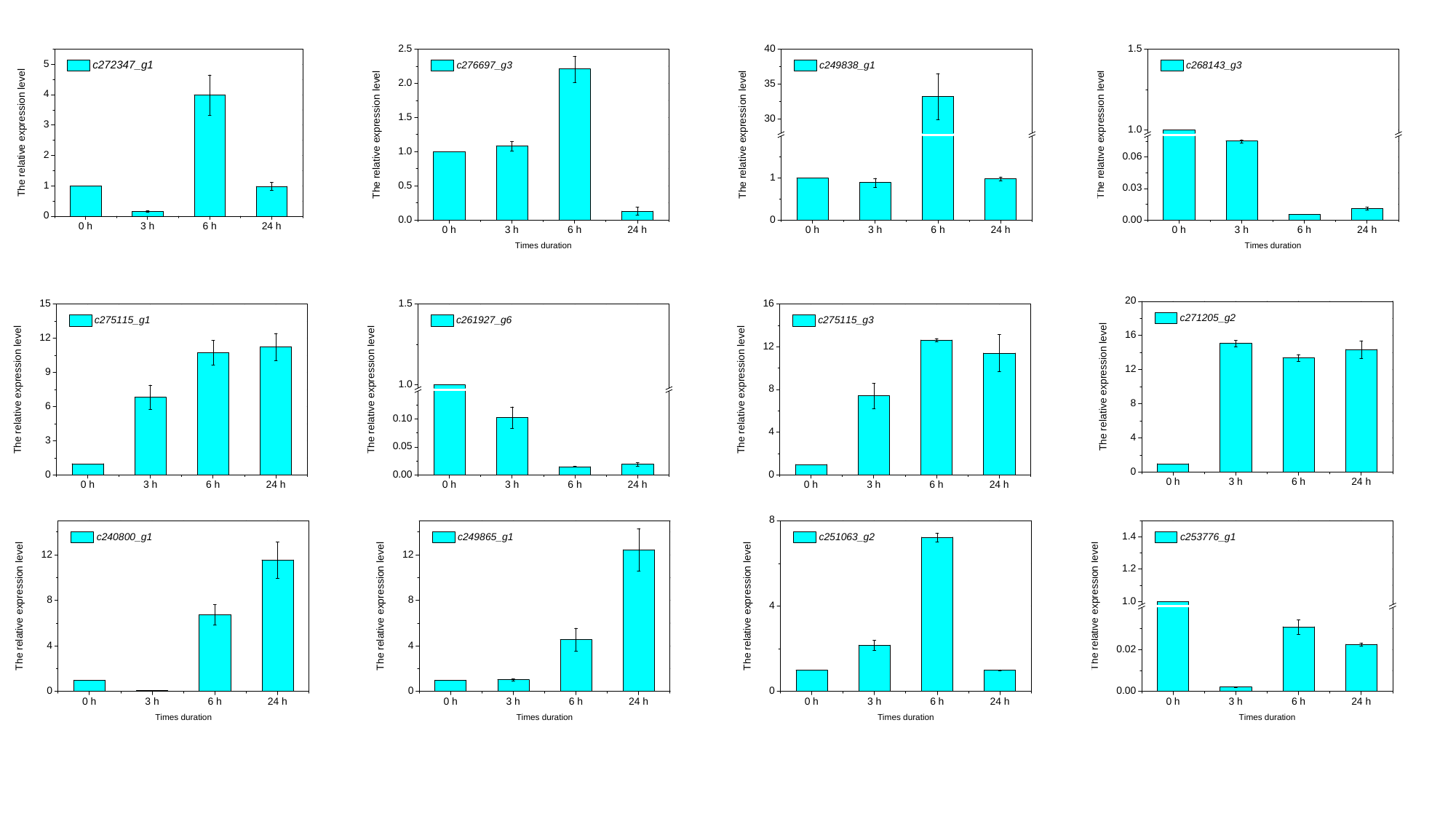

Supplement: Supplementary file 7 — Real-time PCR validation of the tested genes expression. (PPT 352 kb) [file 12864_2017_4055_MOESM7_ESM.ppt]

## Slide 1
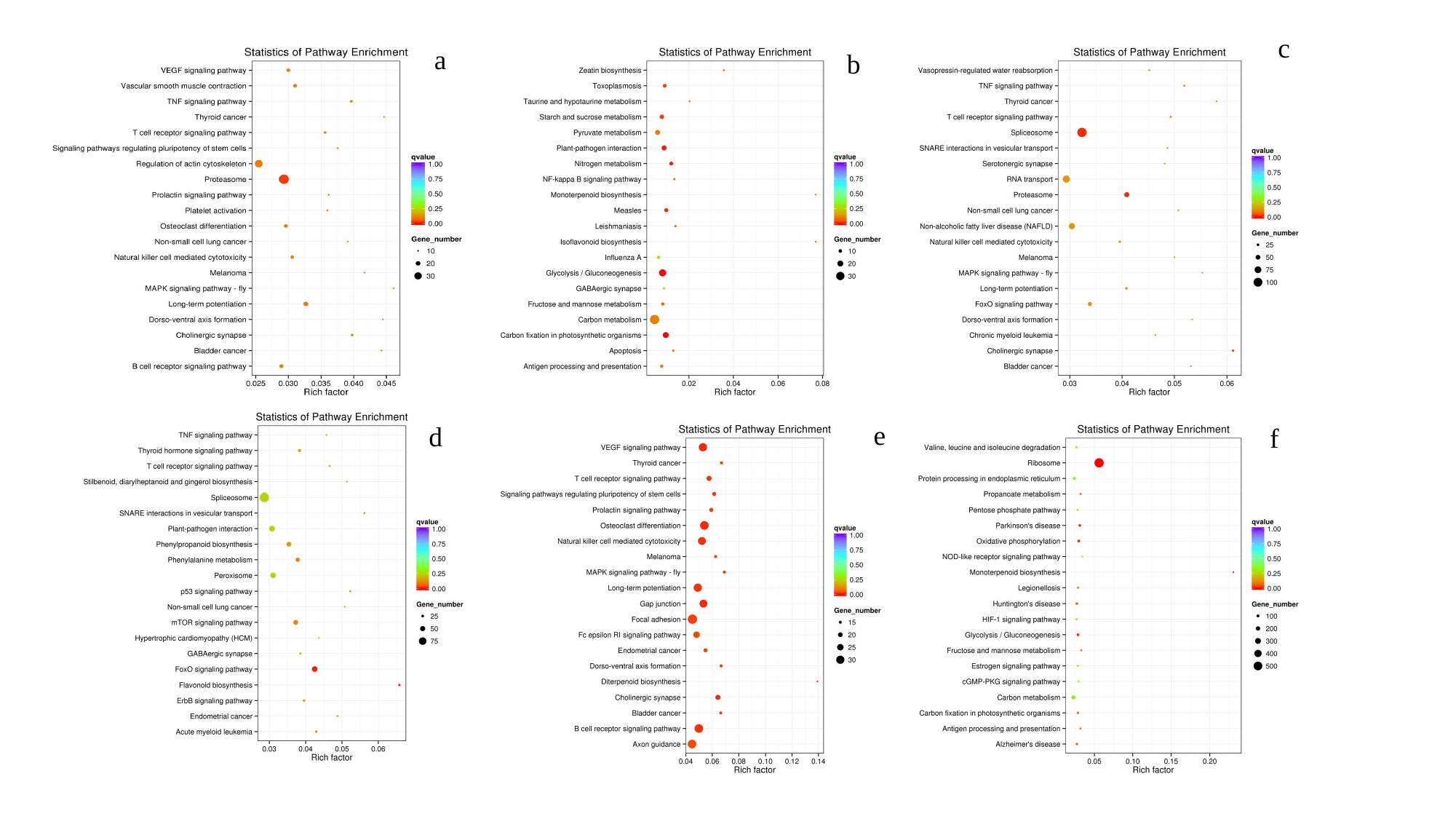

c
a
b
e
d
f

Supplement: Supplementary file 9 — Scatter plot of KEGG pathway enrichment statistics and the most enrichment pathway at different waterlogging durations. a-f: Top 20 statistics of up-regulated and down regulated pathway enrichment at different waterlogging durations. (PPTX 2794 kb) [file 12864_2017_4055_MOESM9_ESM.pptx]
