# Supplementary material for: Physiological and de novo transcriptome analysis of the fermentation mechanism of Cerasus sachalinensis roots in response to short-term waterlogging
Source: BMC Genomics. 2017 Aug 22;18:649. doi: 10.1186/s12864-017-4055-1 (PMC5568329; doi:10.1186/s12864-017-4055-1)
Supplement: Supplementary file 6 — Changes in the expression of genes in sucrose and fermentation pathways of C. sachalinensis under waterlogging stress. (PPT 540 kb) [file 12864_2017_4055_MOESM6_ESM.ppt]

## Slide 1
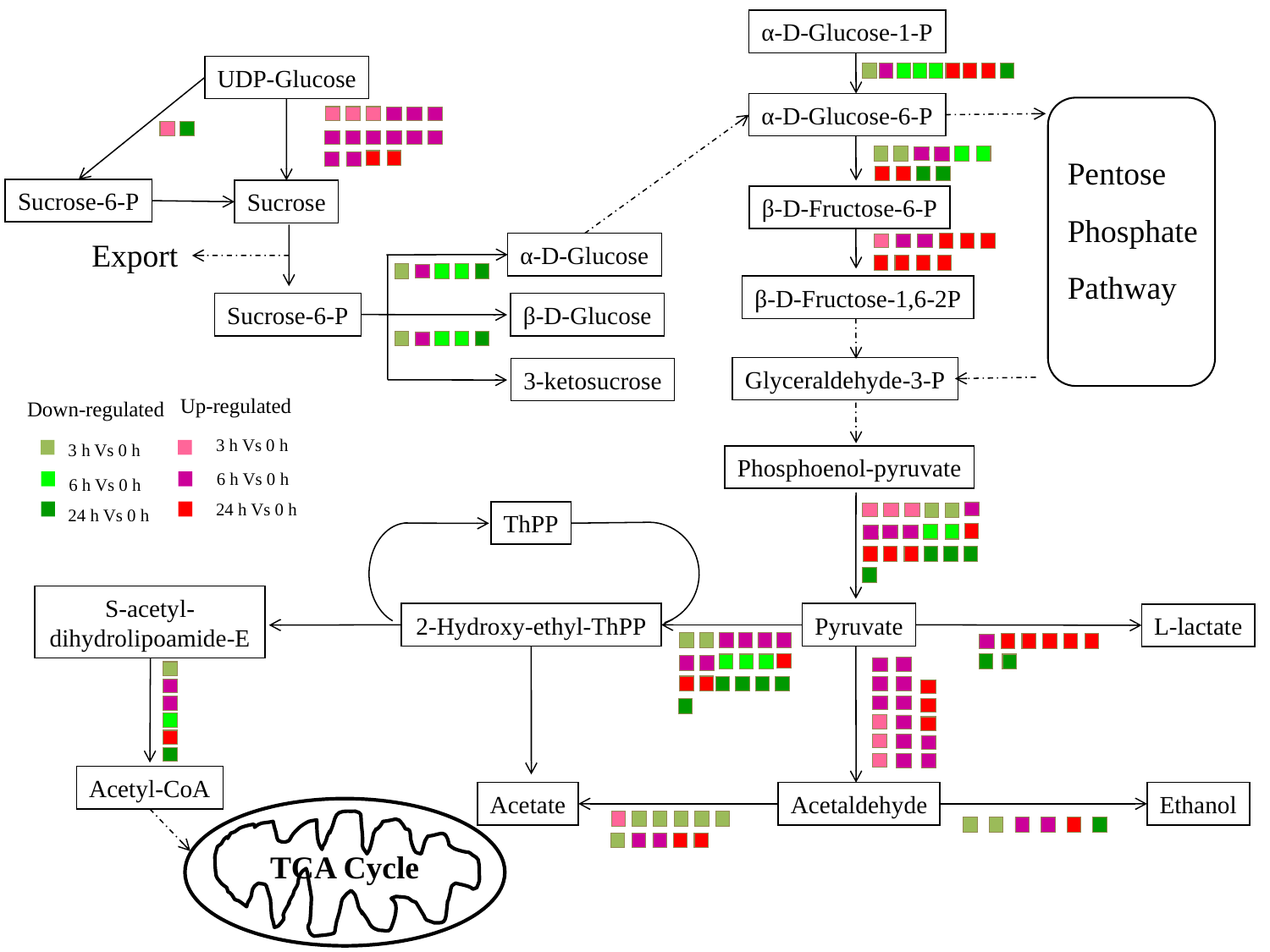

α-D-Glucose-1-P
UDP-Glucose
α-D-Glucose-6-P
Pentose
Phosphate
Pathway
Sucrose-6-P
Sucrose
β-D-Fructose-6-P
Export
α-D-Glucose
β-D-Fructose-1,6-2P
Sucrose-6-P
β-D-Glucose
Glyceraldehyde-3-P
3-ketosucrose
Up-regulated
Down-regulated
3 h Vs 0 h
3 h Vs 0 h
6 h Vs 0 h
6 h Vs 0 h
24 h Vs 0 h
24 h Vs 0 h
Phosphoenol-pyruvate
ThPP
S-acetyl-dihydrolipoamide-E
2-Hydroxy-ethyl-ThPP
Pyruvate
L-lactate
Acetyl-CoA
Acetate
Acetaldehyde
Ethanol
TCA Cycle
